# Ethyl 4-fluoro-3-nitro­benzoate

**DOI:** 10.1107/S1600536809005224

**Published:** 2009-02-21

**Authors:** Shivanagere Nagojappa Narendra Babu, Aisyah Saad Abdul Rahim, Hasnah Osman, Ibrahim Abdul Razak, Hoong-Kun Fun

**Affiliations:** a School of Pharmaceutical Sciences, Universiti Sains Malaysia, 11800 USM, Penang, Malaysia; b School of Chemical Sciences, Universiti Sains Malaysia, 11800 USM, Penang, Malaysia; c X-ray Crystallography Unit, School of Physics, Universiti Sains Malaysia, 11800 USM, Penang, Malaysia

## Abstract

In the title compound, C_9_H_8_FNO_4_, C—H⋯O inter­molecular inter­actions form dimers with *R* ^2^_2_(10) motifs. These dimers are arranged into chains parallel to the *b* axis and the chains are stacked down the *c* axis.

## Related literature

For general background, see: Ishida *et al.* (2006 ▶); Rida *et al.* (2005 ▶); Mohd. Maidin, Abdul Rahim, Abdul Hamid *et al.* (2008 ▶). For bond-length data, see: Allen *et al.* (1987 ▶). For related structures, see: Mohd. Maidin, Abdul Rahim, Osman *et al.* (2008 ▶); Li *et al.* (2008 ▶, 2009 ▶). For details of hydrogen-bond motifs, see: Bernstein *et al.* (1995 ▶). For details on the stability of the temperature controller, see: Cosier & Glazer (1986 ▶).
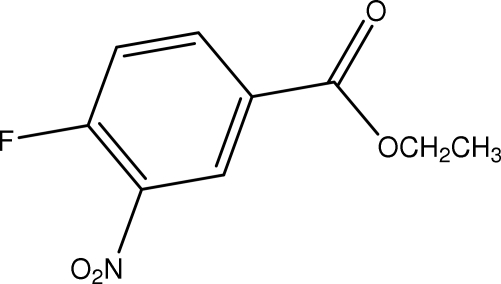


## Experimental

### 

#### Crystal data

C_9_H_8_FNO_4_
*M* *_r_* = 213.16
Monoclinic, 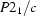

*a* = 9.9246 (3) Å
*b* = 13.2883 (3) Å
*c* = 6.9310 (2) Å
β = 94.410 (2)°
*V* = 911.36 (4) Å^3^
*Z* = 4
Mo *K*α radiation
μ = 0.14 mm^−1^
*T* = 100 K
0.55 × 0.22 × 0.09 mm

#### Data collection

Bruker SMART APEXII CCD area-detector diffractometer
Absorption correction: multi-scan (*SADABS*; Bruker, 2005 ▶) *T*_min_ = 0.929, *T*_max_ = 0.988
12540 measured reflections
2913 independent reflections
2411 reflections with *I* > 2σ(*I*)
*R*_int_ = 0.028

#### Refinement

*R*[*F* ^2^ > 2σ(*F* ^2^)] = 0.045
*wR*(*F* ^2^) = 0.139
*S* = 1.11
2913 reflections
138 parameters
H-atom parameters constrained
Δρ_max_ = 0.55 e Å^−3^
Δρ_min_ = −0.36 e Å^−3^

### 

Data collection: *APEX2* (Bruker, 2005 ▶); cell refinement: *SAINT* (Bruker, 2005 ▶); data reduction: *SAINT*; program(s) used to solve structure: *SHELXTL* (Sheldrick, 2008 ▶); program(s) used to refine structure: *SHELXTL*; molecular graphics: *SHELXTL*; software used to prepare material for publication: *SHELXTL* and *PLATON* (Spek, 2009 ▶).

## Supplementary Material

Crystal structure: contains datablocks global, I. DOI: 10.1107/S1600536809005224/at2723sup1.cif

Structure factors: contains datablocks I. DOI: 10.1107/S1600536809005224/at2723Isup2.hkl

Additional supplementary materials:  crystallographic information; 3D view; checkCIF report

## Figures and Tables

**Table 1:** Hydrogen-bond geometry (Å, °)

*D*—H⋯*A*	*D*—H	H⋯*A*	*D*⋯*A*	*D*—H⋯*A*
C1—H1*A*⋯O4^i^	0.93	2.44	3.2380 (15)	144

Symmetry code: (i) 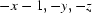.

**Fig. 1.**
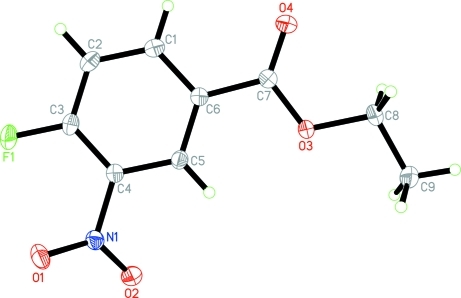
The asymmetric unit of the title compound, showing 50% probability displacement ellipsoids and the atom numbering scheme.

**Fig. 2.**
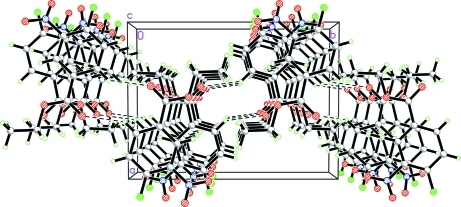
The crystal packing of the title compound, viewed down the c axis. Intermolecular hydrogen bondings are shown as dotted lines.

## supplementary crystallographic information

### Comment

The nitro benzoic acid intermediates are convenient starting materials for the synthesis of various biologically active heterocycles *e.g.* benzimidazoles (Ishida *et al.*, 2006) and benzoxazoles (Rida *et al.*, 2005). As a part of our ongoing studies on new nitro benzoic acid derivatives (Mohd. Maidin, Abdul Rahim, Abdul Hamid *et al.*, 2008), we have synthesized the title compound as an intermediate and report its structure here.

The bond lengths (Allen *et al.*, 1987) and angles observed in (I) are within normal ranges and are consistent with other related structures (Mohd. Maidin, Abdul Rahim, Osman *et al.*, 2008; Li *et al.*, 2009; Li *et al.*, 2008). The C1—H1A···O4^i^ intermolecular interactions (Table 2) linked the molecules into dimers forming 10-membered rings with *R^2^_2_(10)* motifs (Bernstein *et al.*, 1995). In the crystal structure, these dimers are arranged into chains parallel to the *b* axis. The chains are stacked down the *c* axis (Fig. 2).

### Experimental

For the preparation of the title compound, 4-fluoro-3-nitro-benzoic acid (5.0 g, 0.027 mol) was refluxed in absolute ethanol (50 ml) and conc. H_2_SO_4_ (2.0 ml) for 8 h. Upon reaction completion, ethanol was evaporated and the reaction mixture was diluted with water. The aqueous layer was extracted with ethyl acetate (25 *x* 2 ml). The combined organic layer was collected and dried over anhydrous MgSO_4_. The solvent was removed under reduced pressure to afford yellow oil as the crude product. Recrystallization with hot ethyl acetate and petroleum ether (60–80) yielded colourless crystals that were found suitable for X-ray analysis.

### Refinement

All the H atoms were positioned geometrically and refined using a riding model with C—H = 0.93Å for aromatic and 0.96Å for CH_3_. The *U*_iso_ values were constrained to be -1.5*U*_eq_ of the carrier atom for the methyl H atoms and -1.2U_equ_ for the remaining hydrogen atoms. The rotating model group was considered for the methyl group.

### Crystal data

**Table:**

C_9_H_8_FNO_4_	*F*(000) = 440
*M**_r_* = 213.16	*D*_x_ = 1.554 Mg m^−^^3^
Monoclinic, *P*2_1_/*c*	Mo *K*α radiation, λ = 0.71073 Å
Hall symbol: -P 2ybc	Cell parameters from 5027 reflections
*a* = 9.9246 (3) Å	θ = 2.6–30.8°
*b* = 13.2883 (3) Å	µ = 0.14 mm^−^^1^
*c* = 6.9310 (2) Å	*T* = 100 K
β = 94.410 (2)°	Block, colourless
*V* = 911.36 (4) Å^3^	0.55 × 0.22 × 0.09 mm
*Z* = 4	

### Data collection

**Table:**

Bruker SMART APEXII CCD area-detector diffractometer	2913 independent reflections
Radiation source: sealed tube	2411 reflections with *I* > 2σ(*I*)
graphite	*R*_int_ = 0.028
φ and ω scans	θ_max_ = 31.1°, θ_min_ = 2.1°
Absorption correction: multi-scan (*SADABS*; Bruker, 2005)	*h* = −14→14
*T*_min_ = 0.929, *T*_max_ = 0.988	*k* = −18→19
12540 measured reflections	*l* = −10→10

### Refinement

**Table:**

Refinement on *F*^2^	Secondary atom site location: difference Fourier map
Least-squares matrix: full	Hydrogen site location: inferred from neighbouring sites
*R*[*F*^2^ > 2σ(*F*^2^)] = 0.045	H-atom parameters constrained
*wR*(*F*^2^) = 0.139	*w* = 1/[σ^2^(*F*_o_^2^) + (0.0772*P*)^2^ + 0.1955*P*] where *P* = (*F*_o_^2^ + 2*F*_c_^2^)/3
*S* = 1.11	(Δ/σ)_max_ < 0.001
2913 reflections	Δρ_max_ = 0.55 e Å^−^^3^
138 parameters	Δρ_min_ = −0.36 e Å^−^^3^
0 restraints	Extinction correction: *SHELXL*, Fc^*^=kFc[1+0.001xFc^2^λ^3^/sin(2θ)]^-1/4^
Primary atom site location: structure-invariant direct methods	Extinction coefficient: 0.018 (4)

### Special details

**Table:**

Experimental. The crystal was placed in the cold stream of an Oxford Cryosystems Cobra open-flow nitrogen cryostat (Cosier & Glazer, 1986) operating at 100.0 (1) K.
Geometry. All e.s.d.'s (except the e.s.d. in the dihedral angle between two l.s. planes) are estimated using the full covariance matrix. The cell e.s.d.'s are taken into account individually in the estimation of e.s.d.'s in distances, angles and torsion angles; correlations between e.s.d.'s in cell parameters are only used when they are defined by crystal symmetry. An approximate (isotropic) treatment of cell e.s.d.'s is used for estimating e.s.d.'s involving l.s. planes.
Refinement. Refinement of *F*^2^ against ALL reflections. The weighted *R*-factor *wR* and goodness of fit *S* are based on *F*^2^, conventional *R*-factors *R* are based on *F*, with *F* set to zero for negative *F*^2^. The threshold expression of *F*^2^ > σ(*F*^2^) is used only for calculating *R*-factors(gt) *etc*. and is not relevant to the choice of reflections for refinement. *R*-factors based on *F*^2^ are statistically about twice as large as those based on *F*, and *R*- factors based on ALL data will be even larger.

### Fractional atomic coordinates and isotropic or equivalent isotropic displacement parameters (Å^2^)

**Table:**

	*x*	*y*	*z*	*U*_iso_*/*U*_eq_	
F1	0.04351 (7)	0.10679 (6)	0.18019 (12)	0.0241 (2)	
O1	0.07165 (9)	0.29746 (7)	0.29028 (14)	0.0240 (2)	
O2	−0.04092 (9)	0.39394 (6)	0.08249 (15)	0.0247 (2)	
O3	−0.52330 (8)	0.30897 (6)	−0.00372 (14)	0.0203 (2)	
O4	−0.58597 (9)	0.14638 (7)	−0.02358 (16)	0.0276 (2)	
N1	−0.02296 (10)	0.31401 (7)	0.16902 (15)	0.0172 (2)	
C1	−0.31523 (12)	0.08546 (9)	0.06256 (18)	0.0197 (2)	
H1A	−0.3804	0.0356	0.0410	0.024*	
C2	−0.18146 (12)	0.05833 (9)	0.10726 (19)	0.0211 (3)	
H2A	−0.1566	−0.0091	0.1139	0.025*	
C3	−0.08581 (11)	0.13301 (9)	0.14173 (17)	0.0181 (2)	
C4	−0.12280 (11)	0.23409 (8)	0.12960 (16)	0.0154 (2)	
C5	−0.25648 (11)	0.26145 (8)	0.08154 (16)	0.0154 (2)	
H5A	−0.2807	0.3290	0.0710	0.018*	
C6	−0.35338 (11)	0.18648 (8)	0.04945 (17)	0.0161 (2)	
C7	−0.49936 (11)	0.21032 (9)	0.00323 (18)	0.0179 (2)	
C8	−0.66549 (12)	0.33789 (10)	−0.0398 (2)	0.0231 (3)	
H8A	−0.7170	0.3145	0.0648	0.028*	
H8B	−0.7035	0.3081	−0.1597	0.028*	
C9	−0.67127 (13)	0.45039 (10)	−0.0529 (2)	0.0280 (3)	
H9A	−0.7638	0.4716	−0.0713	0.042*	
H9B	−0.6233	0.4725	−0.1603	0.042*	
H9C	−0.6304	0.4791	0.0646	0.042*	

### Atomic displacement parameters (Å^2^)

**Table:**

	*U*^11^	*U*^22^	*U*^33^	*U*^12^	*U*^13^	*U*^23^
F1	0.0168 (3)	0.0239 (4)	0.0312 (4)	0.0077 (3)	0.0003 (3)	0.0020 (3)
O1	0.0153 (4)	0.0295 (5)	0.0264 (5)	0.0006 (3)	−0.0037 (3)	0.0008 (4)
O2	0.0228 (4)	0.0174 (4)	0.0333 (5)	−0.0018 (3)	−0.0025 (4)	0.0042 (4)
O3	0.0123 (4)	0.0155 (4)	0.0324 (5)	0.0009 (3)	−0.0017 (3)	0.0002 (3)
O4	0.0191 (4)	0.0176 (4)	0.0453 (6)	−0.0038 (3)	−0.0041 (4)	0.0012 (4)
N1	0.0141 (4)	0.0180 (5)	0.0196 (5)	0.0007 (3)	0.0017 (3)	−0.0015 (4)
C1	0.0201 (5)	0.0148 (5)	0.0242 (6)	−0.0008 (4)	0.0010 (4)	−0.0002 (4)
C2	0.0229 (6)	0.0140 (5)	0.0263 (6)	0.0037 (4)	0.0015 (5)	0.0004 (4)
C3	0.0166 (5)	0.0185 (5)	0.0192 (5)	0.0049 (4)	0.0015 (4)	0.0006 (4)
C4	0.0144 (5)	0.0158 (5)	0.0159 (5)	0.0007 (4)	0.0010 (4)	0.0001 (4)
C5	0.0144 (5)	0.0149 (5)	0.0169 (5)	0.0018 (4)	0.0014 (4)	0.0005 (4)
C6	0.0148 (5)	0.0152 (5)	0.0181 (5)	0.0005 (4)	0.0004 (4)	−0.0001 (4)
C7	0.0157 (5)	0.0162 (5)	0.0216 (5)	−0.0003 (4)	0.0005 (4)	0.0003 (4)
C8	0.0119 (5)	0.0218 (6)	0.0350 (7)	0.0007 (4)	−0.0013 (4)	0.0022 (5)
C9	0.0188 (6)	0.0213 (6)	0.0426 (8)	0.0040 (4)	−0.0050 (5)	−0.0051 (5)

### Geometric parameters (Å, °)

**Table:**

F1—C3	1.3368 (13)	C3—C4	1.3932 (16)
O1—N1	1.2308 (13)	C4—C5	1.3912 (14)
O2—N1	1.2261 (13)	C5—C6	1.3906 (15)
O3—C7	1.3326 (14)	C5—H5A	0.9300
O3—C8	1.4653 (13)	C6—C7	1.4935 (15)
O4—C7	1.2127 (14)	C8—C9	1.4984 (19)
N1—C4	1.4638 (15)	C8—H8A	0.9700
C1—C2	1.3876 (16)	C8—H8B	0.9700
C1—C6	1.3959 (16)	C9—H9A	0.9600
C1—H1A	0.9300	C9—H9B	0.9600
C2—C3	1.3814 (17)	C9—H9C	0.9600
C2—H2A	0.9300		
			
C7—O3—C8	115.52 (9)	C5—C6—C1	119.85 (10)
O2—N1—O1	124.29 (10)	C5—C6—C7	122.00 (10)
O2—N1—C4	117.80 (9)	C1—C6—C7	118.14 (10)
O1—N1—C4	117.89 (10)	O4—C7—O3	124.15 (11)
C2—C1—C6	120.96 (11)	O4—C7—C6	123.28 (11)
C2—C1—H1A	119.5	O3—C7—C6	112.57 (9)
C6—C1—H1A	119.5	O3—C8—C9	107.69 (9)
C3—C2—C1	119.01 (11)	O3—C8—H8A	110.2
C3—C2—H2A	120.5	C9—C8—H8A	110.2
C1—C2—H2A	120.5	O3—C8—H8B	110.2
F1—C3—C2	118.93 (10)	C9—C8—H8B	110.2
F1—C3—C4	120.52 (10)	H8A—C8—H8B	108.5
C2—C3—C4	120.51 (10)	C8—C9—H9A	109.5
C5—C4—C3	120.56 (10)	C8—C9—H9B	109.5
C5—C4—N1	118.32 (10)	H9A—C9—H9B	109.5
C3—C4—N1	121.11 (10)	C8—C9—H9C	109.5
C6—C5—C4	119.08 (10)	H9A—C9—H9C	109.5
C6—C5—H5A	120.5	H9B—C9—H9C	109.5
C4—C5—H5A	120.5		
			
C6—C1—C2—C3	0.93 (19)	N1—C4—C5—C6	−178.04 (10)
C1—C2—C3—F1	−178.41 (11)	C4—C5—C6—C1	−1.07 (18)
C1—C2—C3—C4	−0.74 (19)	C4—C5—C6—C7	178.12 (10)
F1—C3—C4—C5	177.27 (10)	C2—C1—C6—C5	−0.02 (19)
C2—C3—C4—C5	−0.36 (18)	C2—C1—C6—C7	−179.25 (11)
F1—C3—C4—N1	−3.44 (17)	C8—O3—C7—O4	2.20 (18)
C2—C3—C4—N1	178.93 (11)	C8—O3—C7—C6	−177.35 (10)
O2—N1—C4—C5	−31.36 (16)	C5—C6—C7—O4	−179.29 (12)
O1—N1—C4—C5	146.88 (11)	C1—C6—C7—O4	−0.09 (19)
O2—N1—C4—C3	149.33 (12)	C5—C6—C7—O3	0.26 (16)
O1—N1—C4—C3	−32.42 (16)	C1—C6—C7—O3	179.47 (11)
C3—C4—C5—C6	1.27 (17)	C7—O3—C8—C9	−177.07 (11)

### Hydrogen-bond geometry (Å, °)

**Table:**

*D*—H···*A*	*D*—H	H···*A*	*D*···*A*	*D*—H···*A*
C1—H1A···O4^i^	0.93	2.44	3.2380 (15)	144

Symmetry codes: (i) −*x*−1, −*y*, −*z*.
